# Dynamic maps: a visual-analytic methodology for exploring spatio-temporal disease patterns

**DOI:** 10.1186/1476-069X-8-61

**Published:** 2009-12-30

**Authors:** Denise A Castronovo, Kenneth KH Chui, Elena N Naumova

**Affiliations:** 1 Mapping Sustainability, LLC, Jupiter, FL, USA; 2 Department of Public Health and Community Medicine, Tufts University Medical School, Boston, MA, USA

## Abstract

**Background:**

Epidemiologic studies are often confounded by the human and environmental interactions that are complex and dynamic spatio-temporal processes. Hence, it is difficult to discover nuances in the data and generate pertinent hypotheses. Dynamic mapping, a method to simultaneously visualize temporal and spatial information, was introduced to elucidate such complexities. A conceptual framework for dynamic mapping regarding principles and implementation methods was proposed.

**Methods:**

The spatio-temporal dynamics of *Salmonella *infections for 2002 in the U.S. elderly were depicted via dynamic mapping. Hospitalization records were obtained from the Centers of Medicare and Medicaid Services. To visualize the spatial relationship, hospitalization rates were computed and superimposed onto maps of environmental exposure factors including livestock densities and ambient temperatures. To visualize the temporal relationship, the resultant maps were composed into a movie.

**Results:**

The dynamic maps revealed that the *Salmonella *infections peaked at specific spatio-temporal loci: more clusters were observed in the summer months and higher density of such clusters in the South. The peaks were reached when the average temperatures were greater than 83.4°F (28.6°C). Although the relationship of salmonellosis rates and occurrence of temperature anomalies was non-uniform, a strong synchronization was found between high broiler chicken sales and dense clusters of cases in the summer.

**Conclusions:**

Dynamic mapping is a practical visual-analytic technique for public health practitioners and has an outstanding potential in providing insights into spatio-temporal processes such as revealing outbreak origins, percolation and travelling waves of the diseases, peak timing of seasonal outbreaks, and persistence of disease clusters.

## Background

The interactions between human health and physical environments are complex, for they involve many interacting variables that are geographical, chronological, and demographical. A good understanding of these interactions can lead to better strategized preventions and policies. However, such understanding requires the ability to recognize, track, analyze and represent dynamic spatio-temporal processes [1,2].

Infectious disease surveillance can benefit from the study of dynamic spatio-temporal processes through acquiring more information on three aspects: seasonality of the diseases, synchronization between diseases and exposures, and geographic distribution of diseases and exposures. Many infectious diseases exhibit distinct seasonal trends reflecting a temporal process that alternates between periods of low endemic levels with periods of outbreak. For instance, infection rates due to *Salmonella *spp. or *Campylobacter jejuni *peak in the summer while those due to *Giardia *and *Cryptosporidium *rise in autumn [3]. The temporal synchronizations of water and food-borne disease outbreaks and prevailing weather conditions suggest a strong influence asserted by the environment [4]. Spatially, the seasonal patterns of the diseases and their associations with environmental influences also vary across locations [5]. Understanding of dynamic spatio-temporal processes can organize help to organize the above variables into a systematic framework which would consequently spark new hypotheses. However, these analyses would require an innovative integration of geographic information and time-series data.

When applied to geographic information, visualization refers to the procedure of creating cartographic representations [6], or maps, of spatial data. A dynamic map is a series of "temporally-ordered snapshots of maps that each depicts a period in time" [7]. This tool introduces new insights to researchers into the spatio-temporal processes that would not be possible to demonstrate using static maps or statistical analysis [8,9]. When applied to infectious disease surveillance, dynamic maps show spatio-temporal information of a disease outbreak such as origins, percolation and travelling waves of disease incidence, peak timing of seasonal outbreaks, and persistence of disease clusters. Dynamic maps can also help researchers visually explore relationships through synchronization and overlay of outcome data with exposure variables [6].

The main objective of the study is to introduce dynamic mapping as a method for performing exploratory data analysis of disease dynamics and for generating testable hypotheses. We provide a series of dynamic map examples that overlay *Salmonella*-related hospitalization data for the U.S. elderly (aged 65 and over) onto maps of environmental exposure factors such as livestock, ambient temperature and ambient temperature deviance from the 30-year norm. The secondary objective is to introduce principles for building effective dynamic maps for disease outcome data, which are discussed herein.

## Methods

The methodological description consists of four parts: (i) abstraction and aggregation of the outcome data, (ii) abstraction, aggregation, and mapping of the exposure data, (iii) static mapping of the outcome data, and (iv) composition of the dynamic map. Details of these four steps are as follows.

### Health outcome data--abstraction and aggregation

Hospitalization records for *Salmonella *infections were abstracted from the Centers for Medicare and Medicaid Services (CMS) for all Medicare recipients aged 65 or above in the contiguous U.S. for 2002 (Alaska, Hawaii, Virgin Islands and Puerto Rico were excluded from the analysis). In the U.S., more than 95% of the population aged 65 or above are covered by Medicare; hence the CMS records are nationally representative. Each hospitalization record contains information on the date of admission, ZIP code and state of residence, age and up to 10 diagnostic codes based on the International Classification of Diseases, Ninth Revision, Clinical Modification (ICD-9-CM). Records containing any diagnosis starting with "003", indicating "other *Salmonella *infections", were included in this analysis. Conditions starting with "002", indicating "typhoid and paratyphoid fevers" caused by *Salmonella typhi *infection, were excluded due to their low frequencies.

**Figure 1:**
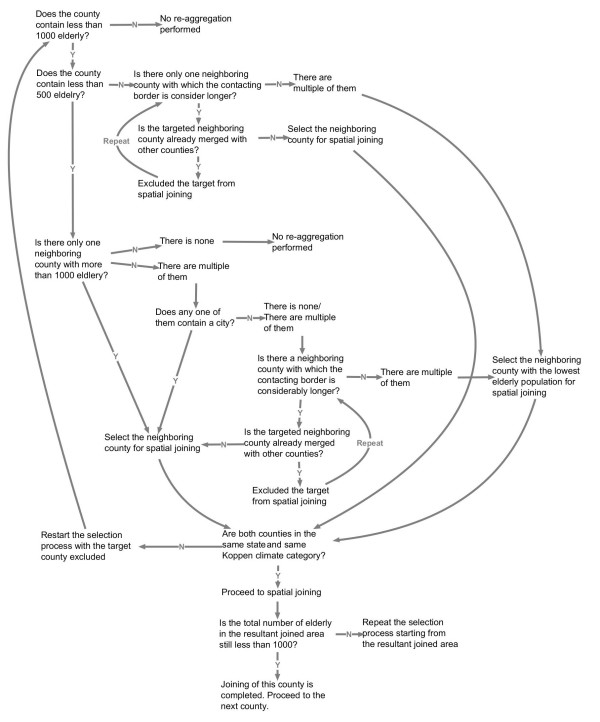
**The re-aggregation scheme**. The chart explains the chain of decisions made when aggregating counties with low elderly population in this study. Arrows with "Y" indicates "Yes"; "N" indicates "No".

Temporally, all records were aggregated to a monthly level; spatially, to a county level. Monthly hospitalization rates were computed using county population aged 65 or above as the denominator. Population counts were obtained from the Census 2000, Summary File 1. To minimize spurious high rates caused by extremely low denominators, a spatial re-aggregation scheme [see Figure 1] was applied to incorporate counties with a low number of elderly into the adjacent counties until the total number of elderly exceeded 1,000, a parameter that was selected based upon the population of elderly per county and the frequency of diseases. The re-aggregation collapsed the number of counties within the continental U.S. from 3,109 to 2,794. On the maps, the boundaries between two aggregated counties were removed to form one larger area. For each aggregated county, the elderly population and monthly *Salmonella *hospitalization counts were summed together. Monthly hospitalization rates per 10,000 elderly, R_*ij*_, were calculated for each aggregated county i (i = 1:2,794) and month j (j = 1:12) as follows:

### Exposure data--abstraction, aggregation and mapping

Ambient temperature data were obtained from the PRISM Group at Oregon State University for average maximum monthly temperature, average maximum annual temperature, and average maximum temperature anomaly for 2002. Data were downloaded in ESRI ArcInfo Ascii Grid format with a spatial resolution of 4 kilometer grid cells and imported into ESRI's ArcMap software. The aggregated county polygons were overlaid onto each temperature grid and the mean temperature or temperature anomaly was calculated for each county. Choropleth maps for the average monthly and annual maximum temperature were created by considering the full range of county-level temperature values with the classification emphasizing temperatures above freezing shown in shades of yellow to dark orange. The monthly temperature anomaly maps show the deviation in temperature on a monthly basis from the 30-year norm for each month. The choropleth maps for the temperature anomaly emphasize temperature deviations greater than +3°C in pink and less than -2°C in purple.

Exposure data on livestock were obtained from the 2002 quinquennial Census of Agriculture gathered by the U.S. Department of Agriculture's National Agricultural Statistics Service. County-level data on the number of broiler and other meat-type chickens sold from livestock farms to food distributors were downloaded and mapped by matching the county FIPS codes with county-level polygons. A choropleth map of the number of broiler chickens sold in 2002 was created by first performing a logarithmic transformation on the data to alleviate the problematic skewness, followed by assigning the data into five categories using a natural breaks classification.

### Mapping of the health outcome

**Figure 2:**
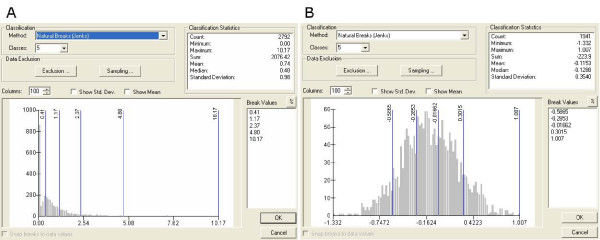
**Natural breaks classification using logarithmic transformation**. A. Natural breaks classification on untransformed *Salmonella *hospitalization rate data, B. Natural breaks classification on log transformed *Salmonella *hospitalization rate data.

Twelve monthly static maps for 2002 were created. To facilitate visual comparison between months, the bin sizes adopted in the legend were unified across the 12 maps. Countywide *Salmonella *hospitalization data for the elderly were extremely skewed due to a disproportionately high amount of low rates and a small amount of high rate outliers. Seasonal changes further exaggerated the problem since there was a two to three-fold increase in salmonellosis during the summer time. To alleviate the problems caused by the skewness, a logarithmic transformation was applied. The logarithmic transformation helps to optimally assign categories into high and low brackets (Figure 2). The unified bin sizes were derived by assigning a natural break classification scheme to the month with the highest amount of hospitalization cases. The resulting classification scheme was then applied to the other maps.

Hospitalization rates were represented on the map with graduated dot symbols. The graduated colour and symbol size of the dots allow for a quick visual comparison between high and low rates; larger and darker dots indicate higher rates and smaller and lighter dots indicate lower rates. The sizes of the graduated dot symbols are proportional to the outcome rates and hence are preferred over polygons with graduating shades, which are harder to interpret due to the wide range of county sizes. The size of the largest graduated dot symbol was determined using the average county size so that overlapping of dots and county boundaries were minimized.

### Dynamic Mapping

The monthly maps of county-level *Salmonella*-related hospitalizations were superimposed onto maps of environmental exposures including: average monthly maximum temperature, the number of broiler chickens sold in 2002, and monthly temperature deviation from the 30-year norm. Maps were exported from ArcMap and imported into Adobe Flash software as an individual key frame. The frames were sequentially incorporated into a movie.

The speed for playing the map movie was optimized so that that the viewers can have enough time to identify clusters and commit the frame to short memory, allowing them to distinguish changes when the next frame appears. Herein, the dynamic maps are shown at a frame rate of one frame per second and the *Salmonella *infection data span two key frames. An interactive interface consisting of control buttons for stop, play, move forward or back one frame, and replay are added to help the viewers investigate the dynamic map at their own pace.

To indicate the calendar time, a label with the year and month was incorporated into the maps. For maps that span more than one year, a sliding calendar bar was added to help the viewer track time duration. The sliding calendar bar could also be interactive so that the viewer could easily navigate to a specific point in time.

Narrations were incorporated into the movies to help the viewers appreciate the dynamics of *Salmonella *hospitalizations with respect to the exposures.

## Results

In 2002, there were a total of 1,686 hospitalizations associated with *Salmonella *infections. The annual rate was 4.85 hospitalizations per 100,000 elderly (aged 65 or above). The infections were more common during the summer and early fall season: 64% of the cases occurred between May and October. For illustrative purposes, Dynamic Map 1 [see Additional file 1] shows the monthly rate of *Salmonella*-related hospitalizations for the U.S. elderly superimposed on a static map of average annual temperature for 2002. The map shows an association between the average-annual temperature gradient, latitude, and longitude. In the Great Plains where elevation is relatively flat there was an overall north to south trend of low to high temperatures. Topography such as higher elevation is also associated with average temperature, as suggested by the Appalachian and Rocky Mountain regions that have lower temperatures. The Northeastern coastal areas experienced warmer temperatures than farther inland.

*Salmonella*-related hospitalizations were most prevalent during the months of July and August, which is consistent with previous studies demonstrating that hot and humid weather is favorable for *Salmonella *survival and replication [3]. The summer is also associated with outdoor cooking where food handling precautions are often inadequate, thereby increasing the risk for individuals to acquire foodborne infections like *Salmonella *[10]. In July, the majority of the hospitalizations occurred in the South, particularly in Mississippi where an apparent cluster existed. One might expect higher counts of salmonellosis in urban areas, but the map demonstrates clusters of low rates in the New York metropolitan area.

Dynamic Map 2 [see Additional file 2] shows the monthly rate of *Salmonella*-related hospitalizations for the U.S. elderly in 2002 superimposed on a dynamic map of average monthly maximum temperature. The map indicates that latitude and longitude are not the best predictors of temperature change due to topography, proximity to the coast, the jet stream and dominant wind patterns. In July, average monthly maximum temperatures less than 90°F (32.2°C) corresponded with higher elevations and predominant wind patterns from the jet stream. *Salmonella *infections peaked during July and August, corresponding with average monthly temperatures greater than 83.4°F (28.6°C).

By overlaying *Salmonella*-related hospitalizations with data of temperature anomaly, as demonstrated in Dynamic Map 3 [see Additional file 3], viewers can determine whether the rates increased when temperatures were significantly higher than the 30-year norm: an indication of a heat wave induced outbreak. Areas on the map shown in orange to pink were warmer than normal and purple areas were colder than normal. In July the cluster of high rates in Mississippi corresponded with temperatures 0 to -3.7°F (0 to -2.1°C) colder than normal. In August, the cluster of salmonellosis in the New York metropolitan area corresponded with temperatures higher than normal of 3.8 to 5.4°F (2.1 to 3.0°C).

Dynamic Map 4 [see Additional file 4] shows the monthly rate of *Salmonella*-related hospitalizations for the U.S. elderly in 2002 superimposed on a map showing the number of broiler chickens sold in 2002 from livestock farms to food distributors. The number of chickens sold per county may be associated with *Salmonella *hospitalizations in the elderly through direct or indirect exposures. The elderly could be directly exposed to *Salmonella *if they work in the chicken industry, or they could be indirectly exposed if the livestock fecal matter contaminates drinking water sources. The dynamic map helps to look for associations between exposure and *Salmonella *incidence. Dynamic Map 4 illustrates that *Salmonella*-related hospitalizations corresponded with high numbers of broiler chickens sold in July in Mississippi and Maryland, in August in North Carolina, and in October in Oklahoma.

## Discussion

### The features of dynamic maps

Dynamic maps provide additional insight into temporal disease dynamics that is not obtainable from static maps. In an exploratory context, static maps can be used to identify and compare patterns in a spatial context only. Static maps allow us to determine what patterns exist, where they exist, how they exist, and identify how they compare to other spatial patterns. Dynamic mapping deepens our understanding of the data by adding the temporal domain [6,11], through which researchers can further investigate when and where diseases emerge, when and to where they spread, for how long or where they persist, and identify differences in their spatio-temporal patterns. Percolation of *Salmonella *infections was demonstrated in the dynamic maps where *Salmonella*-related hospitalizations showed up in northern Mississippi in May and June and spread to southern Mississippi in July. A persistent cluster of *Salmonella*-related hospitalizations occurred in the New York metropolitan area from May through October with an apparent peak in August.

Dynamic maps allow us to investigate processes, such as disease dynamics, rather than simply recognize static patterns: it is useful for modeling disease transmission, place of exposure and probabilities of hospitalization. By overlaying disease outcome with exposure variables, multiple data sets can be integrated to identify trends, degree of synchronization [6] between exposures and outcomes, or even synchronization between two outcomes such as mortality and co-morbidity. Furthermore, dynamic mapping allows for the visualization of disease incidences and seasonal patterns at conventional temporal scales such as daily, weekly or monthly, which would be difficult to convey through traditional static maps. Dynamic Map 2 [Additional file 2] illustrates the seasonal peaks in temperature during the summer months with the hottest temperatures occurring in the South and moving northward. In concurrence with the temperature upsurge, *Salmonella *hospitalizations spread northward from July to August, demonstrating a travelling wave with a potentially strong influence from environmental factors such as temperature and humidity.

Studies have shown that peaks in ambient temperature correspond with peaks in *Salmonella *incidence with a lag of one to five weeks [4,5]. By superimposing monthly *Salmonella *hospitalizations onto maps of ambient temperatures and livestock abundances, dynamic maps can be used to generate hypotheses about routes of exposure, spatial patterns of transmission, and diseases clustering. When viewing the dynamic maps of monthly *Salmonella *infections superimposed onto monthly temperatures the rationale for exploration of a lagged relationship between exposure and outcome emerges. If a lag relationship does exist, dynamic maps offer channel to observe the synchronization between the outcome and exposure.

Dynamic maps allow viewers to track multiple locations of disease occurrence simultaneously. The U.S. Centers for Disease Control and Prevention (CDC) reported minor outbreaks of *Salmonella *during 2002. From January through April of 2002, there were 47 cases of multi-drug resistant *Salmonella *Newport in five states: New York, Michigan, Pennsylvania, Ohio and Connecticut, causing 17 hospitalizations [12]. From March through May of 2002, tainted cantaloupe from Mexico caused 58 cases of *Salmonella *Serotype Poona infections in 10 states: California, Washington, Oregon, Colorado, Nevada, Missouri, Texas, Arkansas, Minnesota, and Vermont. Eleven cases were in individuals aged > 60 years and there were 10 hospitalizations reported [13]. In June 2002, an outbreak of *Salmonella *Serotype Javiana infected people who attended an athletic competition in Orlando, Florida [14]. There were 141 ill people dispersed across 32 states with 3 known hospitalizations. These outbreak examples illustrate that one strain of *Salmonella *may result in a multistate outbreak that affects nonadjacent states. Also, the outbreak may originate in one location but be reported in multiple states depending upon where the infected patient lives. Dynamic maps provide users with a visual-analytic capability that allows more careful consideration of the nuances within the data.

It is important to recognize that the major strength of the proposed dynamic mapping methodology is its visual-analytic capabilities to assist as an exploratory tool to catalyze the scientific process by propagating potential research questions and future directions. While the methodology is not yet ready to test hypotheses, this capability would be the next step for methodological development.

### Designing dynamic maps

Determining class breaks for dynamic maps poses a significant challenge because a single scheme must work for multiple maps and the placement of a class break can either emphasize small fluctuations in the data or mask large changes [15]. Monmonier [7] uses a bin scoring method to assign category breaks to dynamic spatio-temporal choropleth maps in order to avoid creating a visually noisy and constantly changing display that could confound the visualization of major trends in the data. Harrower [15] puts forth a methodology that avoids classification altogether by placing the highest and lowest values at each end of a continuous colour ramp and placing the remaining data proportionally along that continuum for each map frame. His study shows that this unclassed choropleth dynamic mapping method can improve our ability to see gradual changes in the mapped data which more realistically reflect the underlying process. After the class break schema is determined, dynamic maps are built by producing individual map frames one-by-one for each point in time [16] using GIS and animation software [17].

When we developed our principles for effective dynamic mapping, we heavily borrowed from Harrower's [18] principles including: (1) create a simple base map with only a few data classes or features so that it is quickly readable, (2) provide map controls to stop and playback the movie frame-by-frame, (3) direct the viewer's attention with voice over or dynamic map symbols, (4) generalize the map data to highlight important trends, (5) use categorical data legends such as "low", "medium" and "high", and (6) provide a short introduction to the interface before showing the data to break up the learning curve.

### Seven principles for effective dynamic mapping

Many factors should be considered when composing effective dynamic maps for disease incidence data. The following seven principles were suggested for consideration.

#### Principle 1

Choose the optimal aggregation scheme. One important consideration for dynamic mapping of seasonal disease outcome data is to select a temporal scale that is small enough to pick up the seasonal signal while large enough to be easily compiled. Choosing a temporal scale that is too small could cause too many sporadic changes in the dynamic maps that would prevent the map reader from seeing the overall seasonal trend, especially since the viewers' visual memory from one frame to the next is extremely limited. In the examples, countywide *Salmonella*-related hospitalization counts for the elderly were so low that the data had to be aggregated to a monthly level to show seasonal patterns and reduce noise.

The dynamic mapping methodology is best suited for temporal data with a high frequency such as daily, weekly, or monthly occurrences. With each level of data aggregation there is a trade-off between what is gained in showing overall trends and what is lost in terms of visualizing the dynamic properties of the disease (e.g. outbreak origins, persistence, percolation, travelling waves, and peak timing). Recognizing this trade-off is especially important when studying the spatio-temporal dynamics of highly communicable diseases or diseases with very short incubation periods, such as influenza. Researchers should optimize the spatio-temporal scale according to their expertise.

#### Principle 2

Choose the correct colour scheme and legends. For the temporal information to be efficiently transferred, cartographers should minimize the viewers' effort on locating and comparing the key information on the dynamic maps. For instance, adding a dark background colour behind the map helps the user focus on the data represented by the brighter colours. The classification schemes used in the legend should also be unified so that a certain hue, saturation, brightness, transparency, and size represent the same information across the frames in a set of dynamic maps. When overlaying health outcome data onto exposure data, the colour scheme for outcome should be distinguishable from that of the exposure data. Complimentary colours (colours that are opposite to each other on a colour wheel) work well with the exception of reds and greens, which are not distinguishable among viewers with colour-blindness. Colour gradations need to be different enough so that each one is distinguishable at a quick glance. By changing the colour saturation and value, colours are easily made lighter or darker within the same hue.

#### Principle 3

Choose the correct frame speed. In this study the frame speed was optimized to 1 frame/second. However, the speed can be adjusted depending on the size of the map and complexity of the data. When viewing the maps, it is best to view them for at least three times. The first viewing focuses on understanding the exposure variable in the background. The second viewing entails understanding the outcome variable and its spatial patterns. In the third round the viewers can look for relationships between exposure and outcome, clusters of health outcomes, and percolation.

#### Principle 4

Think like the viewers. The usefulness of dynamic maps for environmental and public health research depends heavily on the cartographer's ability to make the maps simple, informative and attractive [18]. Morrison [19] and Harrower [18] discussed four challenges associated with watching and learning from dynamic maps: 1) disappearance--the maps change dramatically from moment to moment causing the map readers to miss information, 2) attention--map readers are confused by the map interface or do not know where to look on the map, 3) complexity--many maps have too much information which confounds their message, and 4) confidence--viewers are less confident in the knowledge they acquire from dynamic maps due to inexperience with them. Our *Salmonella *dynamic map examples used several principles identified by Harrower [18] to help overcome these challenges. To overcome issues of disappearance, the maps used a simple base map, colour-blindness friendly colour schemes, and map playback tools that allow the user to watch the animation many times, stop it and continue frame-by-frame. To reduce the complexity of the maps, the monthly data were filtered to include only a few months prior to and after the seasonal peak so that periods with low endemic levels of *Salmonella *infections were not included.

#### Principle 5

Incorporate control interface. Interaction with dynamic maps, such as building in a set of buttons that control the flow of the maps, allows users to pace their learning.

#### Principle 6

Understand the limitations of dynamic maps. Dynamic maps represent a delicate balance between being useful versus incomprehensible from cognitive and perceptual information overload [20,21]. Since the frames of animated maps change frequently, animated maps are useful for gaining an overall perspective of spatio-temporal processes rather than emphasizing specific rates for specific locations and time periods [7,8,18]. According to Harrower [21], 'the problem is not that map viewers are incapable of seeing the changes occurring on the animated map, it is that they have well-documented trouble remembering what they saw and integrating it into their knowledge schemata.' One of the key research challenges for space-time visualization is to understand the types of problems that are best suited for animated maps [21,22].

#### Principle 7

Be open to improvement. Dynamic maps require careful interpretation, especially when they are applied to outcome data with low counts. Visual clusters of hospitalization rates need to be further explored into the potential number of cases because sometimes high rates are created from single hospitalizations in areas with a small elderly population. The CDC defines an outbreak of foodborne illness as a cluster of two or more infections caused by the same pathogen and linked to the same food [23]. In this study, we attempted to alleviate this problem by introducing the re-aggregation scheme. The versatile functions in GIS software should be thoroughly explored and implemented in order to compensate inherent shortcomings in the data that can potentially mislead the viewers.

## Conclusion

Dynamic mapping provides a practical visualization tool to examine seasonality in infectious disease and its relationship to environmental factors. This paper applied the approach to study *Salmonella*-related hospitalizations nationwide in the U.S. elderly population for the 2002 season and demonstrated a framework that can stimulate hypothesis generation for better understanding disease seasonality. As an extension of our earlier work [24], fundamental principles for making effective dynamic maps were proposed. These rules serve as an intuitive guideline for properly constructing a graphical display that contains a well-understood statistical context or logical path, helping to explain data or concepts by taking advantage of visual perceptions, and objectively displaying the results and concepts in a manner that highlights the unexpected and motivates questions.

Understanding the nature of environmental exposures, the temporal features of disease manifestation, and the linkages between health outcomes and the environment can be substantially enhanced by modern visual-analytical tools. Research in the fields of environmental epidemiology would benefit from the proposed approach of dynamic mapping where data mining and exploration, hypothesis building and testing are required.

## List of Abbreviations

CDC: Centers for Disease Control and Prevention; CMS: Centers for Medicare and Medicaid Services; FIPS code: Federal Information Processing Standard code; GIS: Geographic Information System; ICD-9-CM: International Classification of Diseases, Ninth Revision, Clinical Modification; PRISM: Preparation for Instruction of Science & Math; U.S.: The United States; ZIP code: Zoning Improvement Plan code

## Competing interests

The authors declare that they have no competing interests.

## Authors' contributions

DAC created the dynamic maps and drafted the manuscript. KKHC drafted the manuscript and created Figures. ENN conceived the study and participated in its design and coordination. All authors read and approved the final manuscript.

## Supplementary Material

Additional file 1 **Dynamic Map 1**. Monthly rate of *Salmonella *hospitalizations for the U.S. elderly ages 65 and over superimposed on the average temperature for the year 2002. Click here for file

Additional file 2 **Dynamic Map 2**. Monthly rate of *Salmonella *hospitalizations for the U.S. elderly ages 65 and over superimposed on the average monthly maximum temperature. Click here for file

Additional file 3 **Dynamic Map 3**. Monthly rate of *Salmonella *hospitalizations for the U.S. elderly ages 65 and over superimposed on the temperature deviation from the 30-year norm. Click here for file

Additional file 4 **Dynamic Map 4**. Monthly rate of *Salmonella *hospitalizations for the U.S. elderly ages 65 and over superimposed on number of broiler chickens sold in 2002. Click here for file
